# Advanced tools for molecular characterization of bio-based and biodegradable polymers

**DOI:** 10.1007/s00216-024-05255-3

**Published:** 2024-03-22

**Authors:** Ndumiso Sibanda, Helen Pfukwa, Paul Eselem Bungu, Harald Pasch

**Affiliations:** 1 Department of Chemistry and Polymer Science, University of Stellenbosch, Stellenbosch, 7602 South Africa; 2 Department of Correlative Characterization, Institute of Functional Materials for Sustainability, Helmholtz Center Hereon, Kantstrasse 55, 14513 Teltow, Germany

**Keywords:** Multidimensional liquid chromatography, Hyphenated techniques, Molecular structure, Biodegradable polymers, Bio-based polymers

## Abstract

Bio-based and biodegradable materials play a vital role in a sustainable and green economy. These materials must exhibit properties that are similar to or better than the properties of oil- or coal-based materials and require sophisticated synthesis technologies and detailed knowledge of structure–property correlations. For comprehensive molecular structure elucidation, advanced analytical methods, including coupled and hyphenated techniques that combine advanced fractionation and information-rich spectroscopic detectors, are an indispensable tool. One important tool for fractionating complex polymers regarding molecular size is size exclusion chromatography. For fractionating polymers with regard to chemical composition, solvent (or temperature) gradient HPLC has been developed. The combination of different liquid chromatography methods in comprehensive two-dimensional HPLC setups is another important tool. Today, a toolbox of HPLC methods is in place that enables the fractionation of complex bio-based and biodegradable polymers according to the most important molecular parameters including molecular size, composition, functionality, and branching. Here, an overview of the different techniques and some major applications is presented. Some representative developments in the field are discussed, and different techniques, experimental protocols, and applications are highlighted.

**Graphical Abstract:**

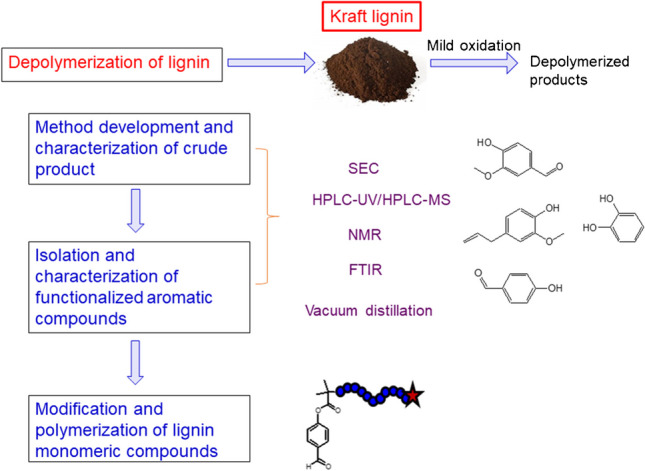

## Introduction

In a sustainable and green economy, bio-based and biodegradable materials play a vital role. No matter if these materials are used in automotive or packaging industries, in pharmaceutics, or regenerative medicine, they must exhibit properties that are similar or better than the properties of oil- or coal-based materials. This requires sophisticated synthesis and production technologies and a deep understanding of structure–property correlations. An indispensable tool for comprehensive molecular structure elucidation are advanced analytical methods, including coupled and hyphenated techniques that combine advanced fractionation with powerful spectroscopic analysis.

**Fig. 1:**
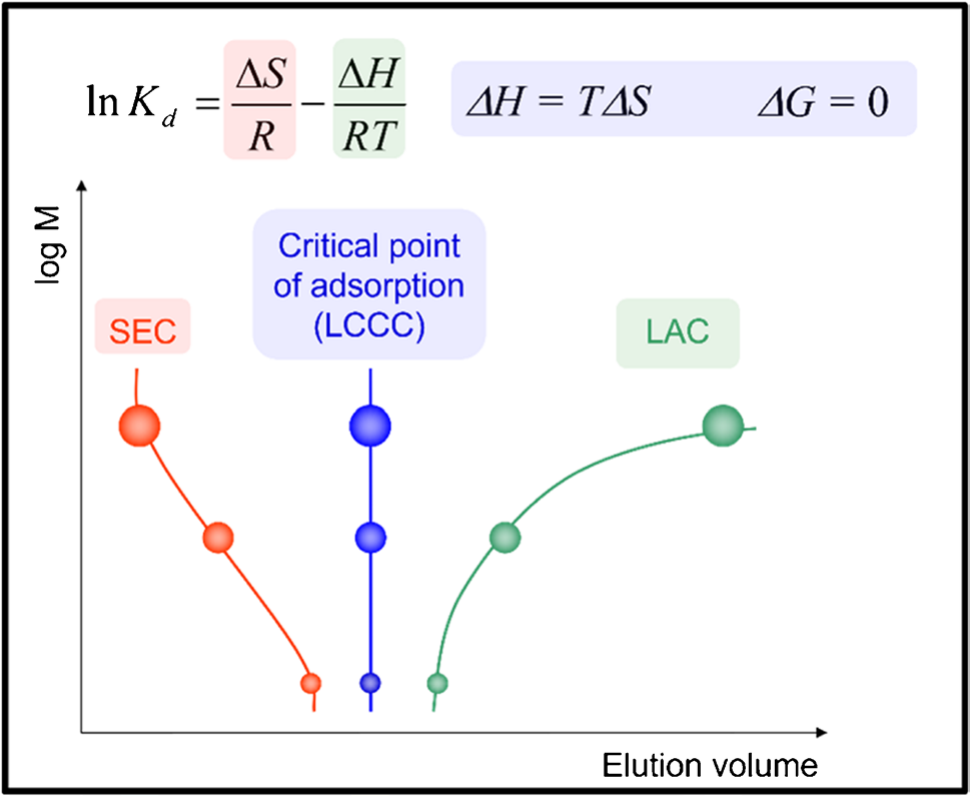
Molar mass dependence of retention in different modes of polymer chromatography; in all cases, the chromatographic behavior is determined by the interplay of enthalpic and entropic effects, and temperature

For green organic polymers, the most important molecular parameters are molar mass and molecular size, chemical composition, the type and distribution of functional groups, and the molecular topology/architecture. All molecular parameters are distributed properties and, accordingly, the comprehensive analysis of green organic polymers requires fractionation [1, 2]. There are well-established and novel fractionation methods that include size exclusion chromatography (SEC) for molar mass separation, liquid adsorption chromatography (LAC) for chemical composition separation, and liquid chromatography at critical conditions (LCCC) for the separation regarding functional groups [1–3]. A schematic presentation of the retention behavior of homopolymers as a function of molar mass as influenced by enthalpy (ΔH), entropy (ΔS), and temperature (T) is presented in Fig. 1.

In SEC, entropic effects are dominating while LAC is dominated by enthalpic effects. In LCCC, entropic and enthalpic effects compensate each other and in this case retention is not influenced by molar mass but parameters like chemical composition or functionality.

**Fig. 2:**
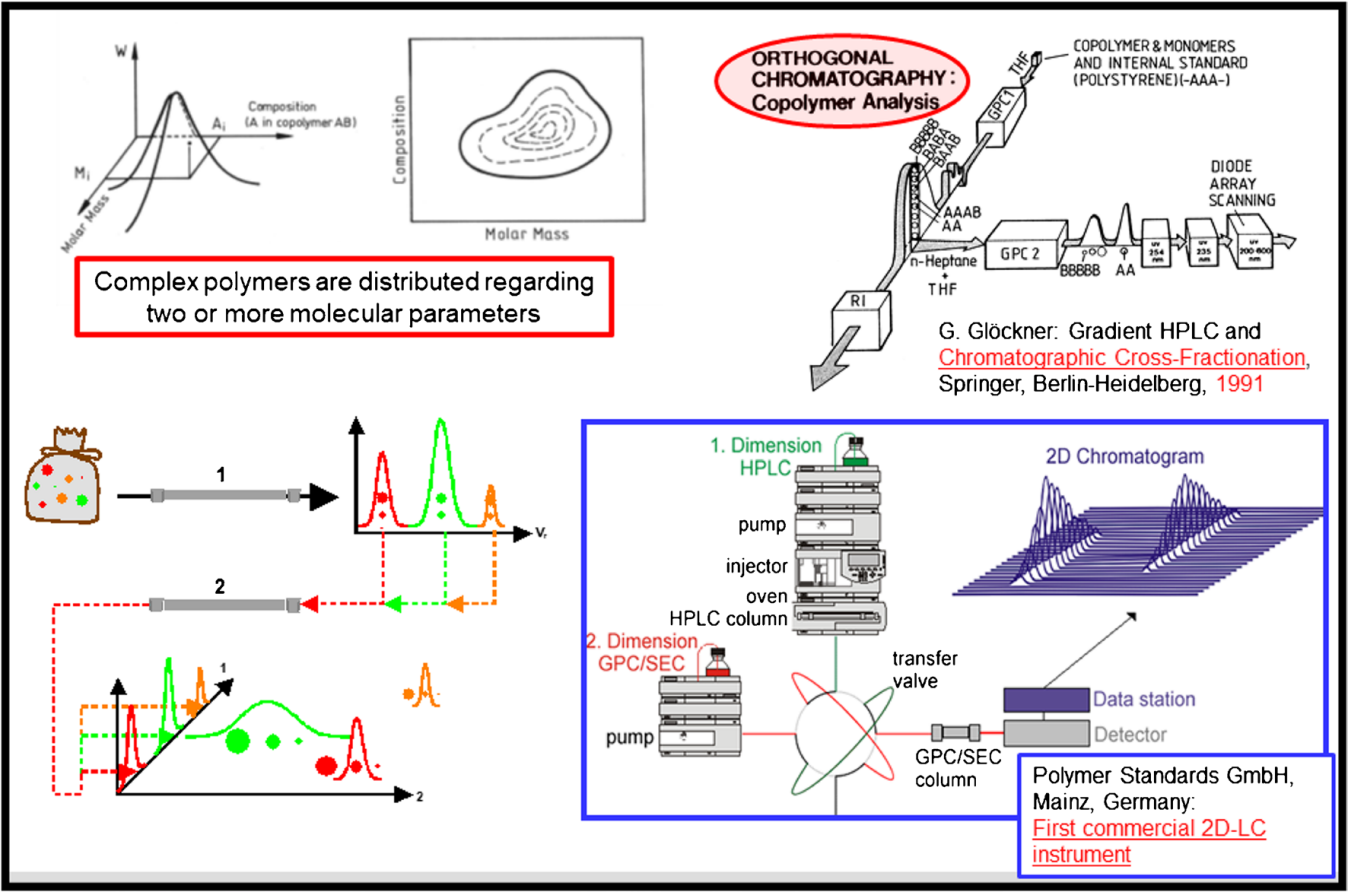
Schematic presentation of comprehensive two-dimensional chromatography for the analysis of complex polymers

As complex polymers are typically distributed in more than one molecular parameter, e.g., molar mass and chemical composition, comprehensive fractionation must be conducted in two orthogonal directions by combining different LC mechanisms. For copolymers, LAC (1st dimension) is combined with SEC (2nd dimension) and the complex sample is separated with regard to chemical composition in the 1st dimension [1, 4–6]. Chemically homogeneous fractions are transferred to the 2nd dimension where separation takes place with regard to molar mass (see schematic presentation in Fig. 2). The first commercial instrument for comprehensive two-dimensional liquid chromatography (2D-LC) of polymers was introduced by Polymer Standards Service GmbH, Germany [7].

**Fig. 3:**
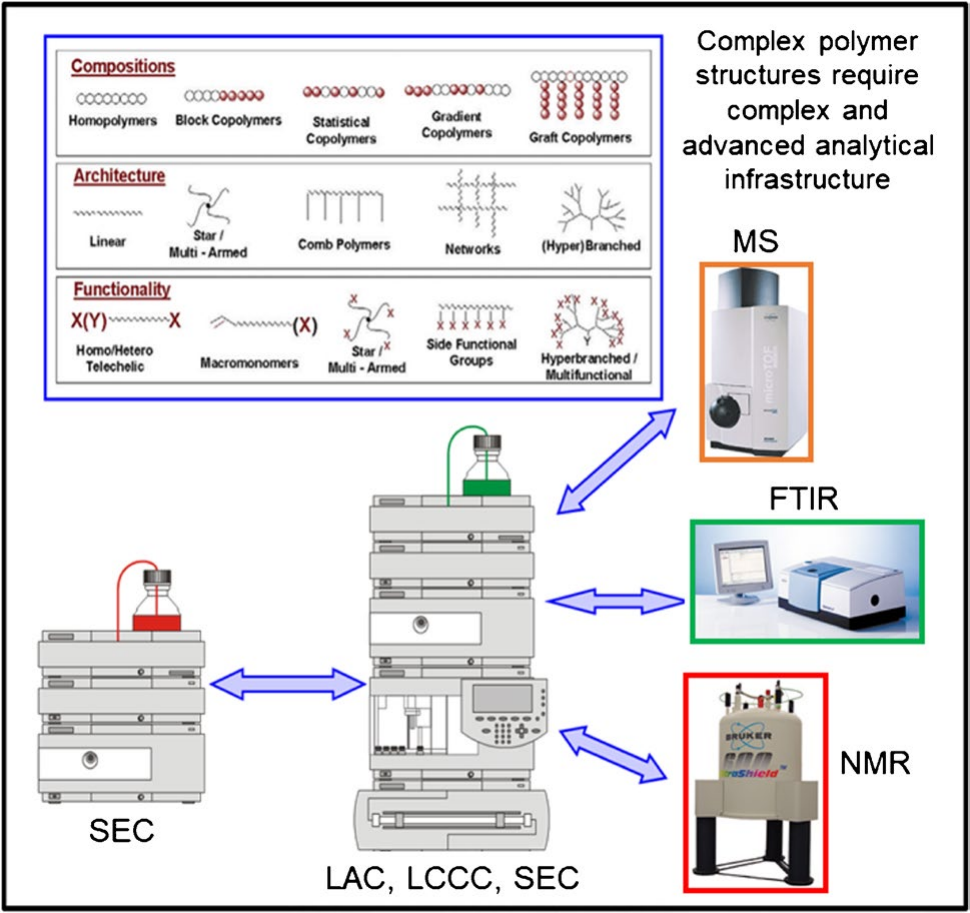
The “LEGO approach” for the analysis of complex polymers based on the combination of different liquid chromatographic methods and information-rich spectroscopic detectors

In addition to selective fractionation, the precise identification and analysis of LC fractions is a necessity. This can be done by direct coupling of spectrometric detectors, such as mass spectrometry, nuclear magnetic resonance, or infrared spectroscopy to LC instruments [8–13]. Such method combinations can address all possible molecular parameters of polymers and can be regarded as the “LEGO approach” to polymer analysis (see Fig. 3).

It has been shown in numerous applications that the comprehensive analysis of bio-based and biodegradable polymers requires the use of various method combinations. In the following sections, a few representative examples of the “LEGO approach” are reviewed.

## Comprehensive analysis of modified hyaluronic acid

Hyaluronic acid (HA, hyaluronan) is an attractive polymer for advanced bio-based materials that exhibits properties such as stimuli responsiveness, biocompatibility, and biodegradability [14–17]. HA has a linear structure and consists of a repeating disaccharide unit comprising β-(1–4)-d-glucuronic acid and β-(1–3)-N-acetyl-d-glucosamine. Modification of HA typically takes place at its four hydroxyl groups since alteration of the carboxyl group can affect its biological properties [18]. Various reactions such as amidation, esterification, etherification, and oxidation are used. The chemical heterogeneity of HA is classified as (1) degree of substitution (DS), i.e., the average number of substituents per HA repeat unit; (2) 1st-order heterogeneity, i.e., the distribution of substituents among the polymer chains; and (3) 2nd-order heterogeneity, i.e., the distribution of substituents along the polymer chain. NMR spectroscopy provides average chemical composition information on modified HA [19, 20]. Substituted HA typically exhibits DS ranging from 0 (no substitution) to 4 (100% substitution). Separation of such complex samples according to DS is an important experimental protocol for comprehensive analysis.

**Fig. 4:**
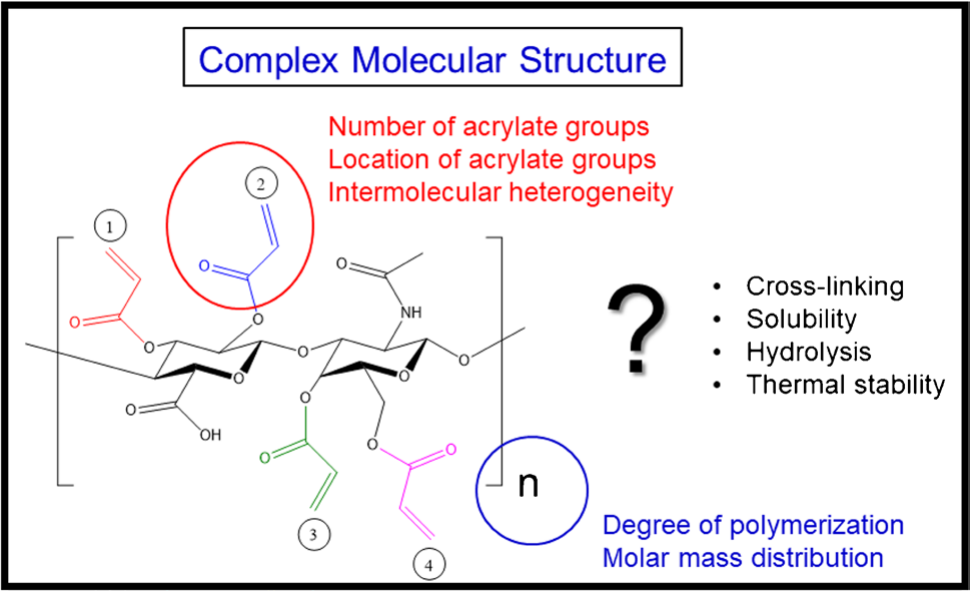
Molecular structure of acrylate-modified HA and related problems that must be addressed

The present work aims at investigating the molecular structure of HA and its acrylate derivatives by HPLC. A chromatographic technique for the separation of HA and its derivatives and the characterization of HAs according to DS over the complete DS range of 0 to 3.4 is addressed. For these sensitive analytes, sample treatment is an important issue as cross-linking and hydrolysis may occur (Fig. 4).

The characterization of HA and its derivatives has been reported previously. Different investigations focused on using liquid chromatography [21–24], FTIR spectroscopy [25], NMR spectroscopy [26], and matrix-assisted laser desorption/ionization mass spectrometry (MALDI-MS) [27] techniques that, however, only characterize the polymer according to a single property such as composition or molar mass. These single methods do not yield sufficient information on the correlation between molar mass and composition (DS) distributions.

**Fig. 5:**
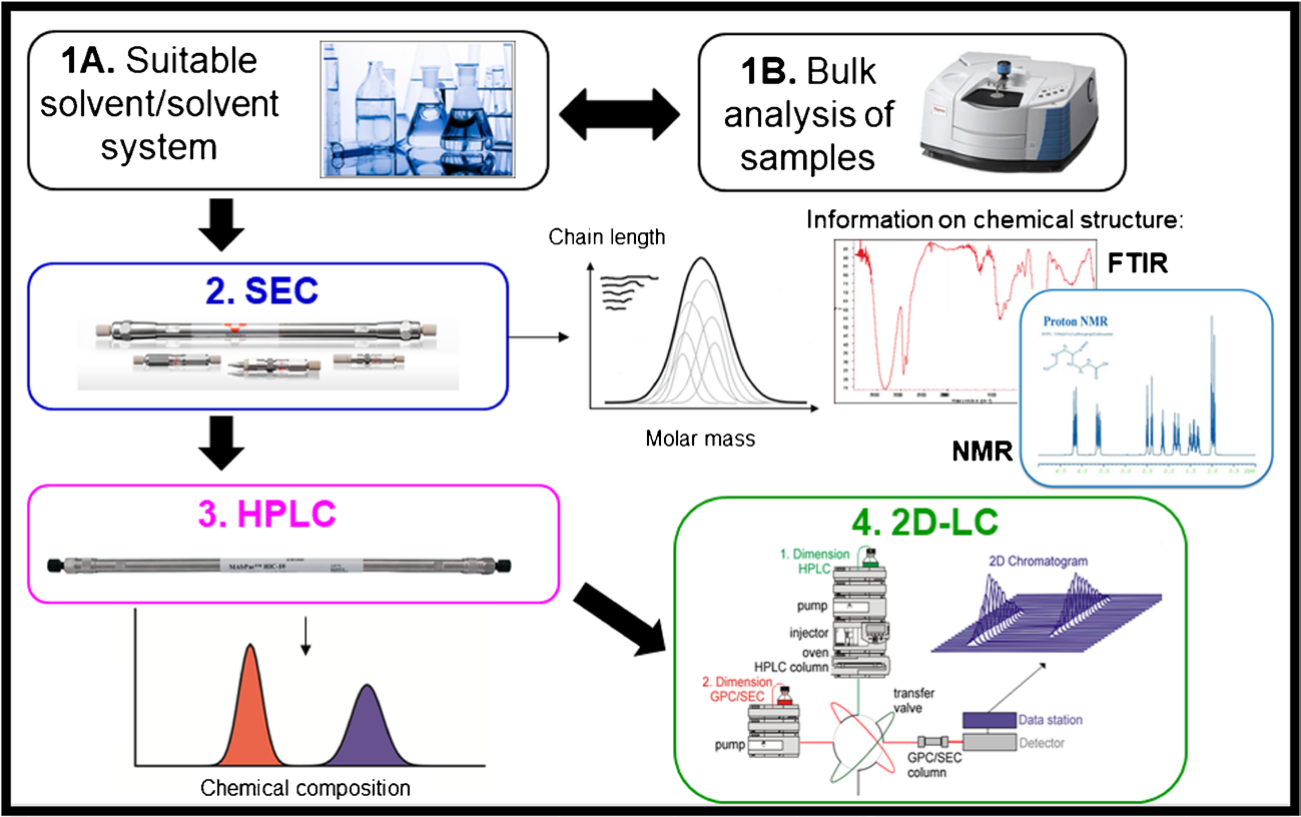
Analytical protocol and suitable techniques for the analysis of modified HA

The analytical approach to the comprehensive characterization of modified HA is schematically presented in Fig. 5. After the investigation of the samples’ solubility and the identification of suitable solvents and mobile phases for SEC and HPLC, bulk analysis is conducted by FTIR and NMR. This is followed by chromatographic method development aiming at analyzing the chemical composition and molar mass of the samples. Ultimately, chemical composition and molar mass are correlated using comprehensive 2D-LC.

The investigation of the samples’ solubility was conducted at temperatures of 25 and 40 °C as it was found that at higher temperatures sample degradation takes place. In total, 35 solvent combinations were tested (including different solvents and variations in solvent ratios) and optimum solubility for both HA and the modified samples was obtained in DMSO-water. In a series of SEC experiments, it was found that precise and reproducible molar mass analysis corresponded to the following experimental conditions: stationary phase: polyester-based GRAM, mobile phase: DMSO-water 60:40 (v/v) plus 0.05 M LiBr, operating temperature 40 °C [28]. With such experimental conditions, however, one must be careful since preferential solvation may occur. In their investigations, the authors varied the LiBr concentration to find the best conditions where this effect is reduced to a minimum.

**Fig. 6:**
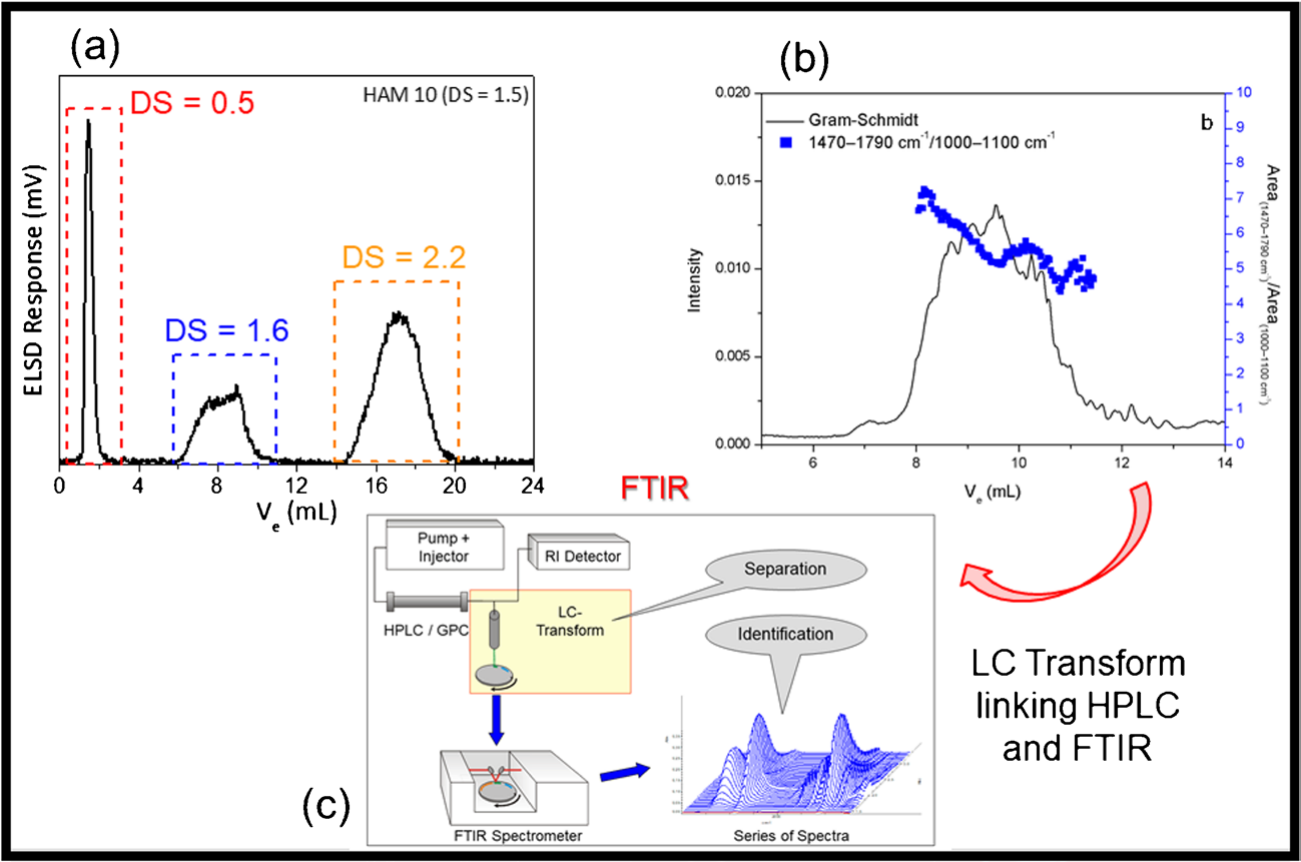
Separation of acrylate-modified HA according to degree of substitution (DS): (**a**) separation in the direction of increasing DS using C_8_-modified silica and ACN-water-NH_4_Ac, (**b**) separation in the direction of decreasing DS using cyano-modified silica and ACN-water, (**c**) schematic presentation of LC-FTIR coupling using the LC Transform system

In the next step of the research, HPLC method development has been conducted [29]. Based on the amphiphilic structure of the analytes, separation could be achieved based on the polarity or the hydrophobicity of these molecules. In a first approach, reversed phase solvent gradient HPLC was explored, and separation was based on the interaction of the hydrophobic acrylate moieties with a non-polar C8 stationary phase. The separation was optimized by using an electrolyte in the sample solvent to suppress non-covalent interactions and improve the selectivity of the developed method. In the second approach, normal phase solvent gradient HPLC was employed and separation was based on the interactions of HA’s polar hydroxyl groups with a polar cyano-modified stationary phase. Some representative results are summarized in Fig. 6.

In agreement with the two experimental protocols, separations in the direction of increasing DS (a) and decreasing DS (b) are presented. For case (a), a nonpolar C_8_-modified silica material was used as the stationary phase. The sample was dissolved in ACN/H_2_O (40:60 v/v) with 0.02 M ammonium acetate (NH_4_Ac) and separation was achieved using a stepwise water/ACN gradient. As can be seen, a sample with an average DS of 1.5 was separated into three fractions with DS of 0.5, 1.6, and 2.2 (as determined by NMR). In (b), the separation according to decreased hydrophobicity and, therefore, decreasing DS, is presented. In this case, a cyano-modified silica is used as the normal stationary phase and the mobile phase was a linear ACN-water gradient. To prove the concept, this HPLC experiment was coupled with FTIR using the LC Transform system that is schematically presented in (c) [4]. The trendline in (b) presenting the average carbonyl group concentration (representative for the acrylate groups) shows that with increasing elution volume a decrease in DS is observed.

The modified HAs exhibit significant heterogeneity regarding molar mass and DS as proven by these experiments. Online two-dimensional liquid chromatography (2D-LC) is the method of choice for the comprehensive analysis of such complex multicomponent analytes. Separation according to chemical composition/DS shall be conducted by using a stepwise solvent gradient and a reversed phase C_8_ column in the 1st dimension of the 2D-LC method. Fractions from the 1st dimension will be transferred to the 2nd dimension comprising size exclusion chromatographic separation of the fractions according to molar mass [30] using an automatic mode of operation.

**Fig. 7:**
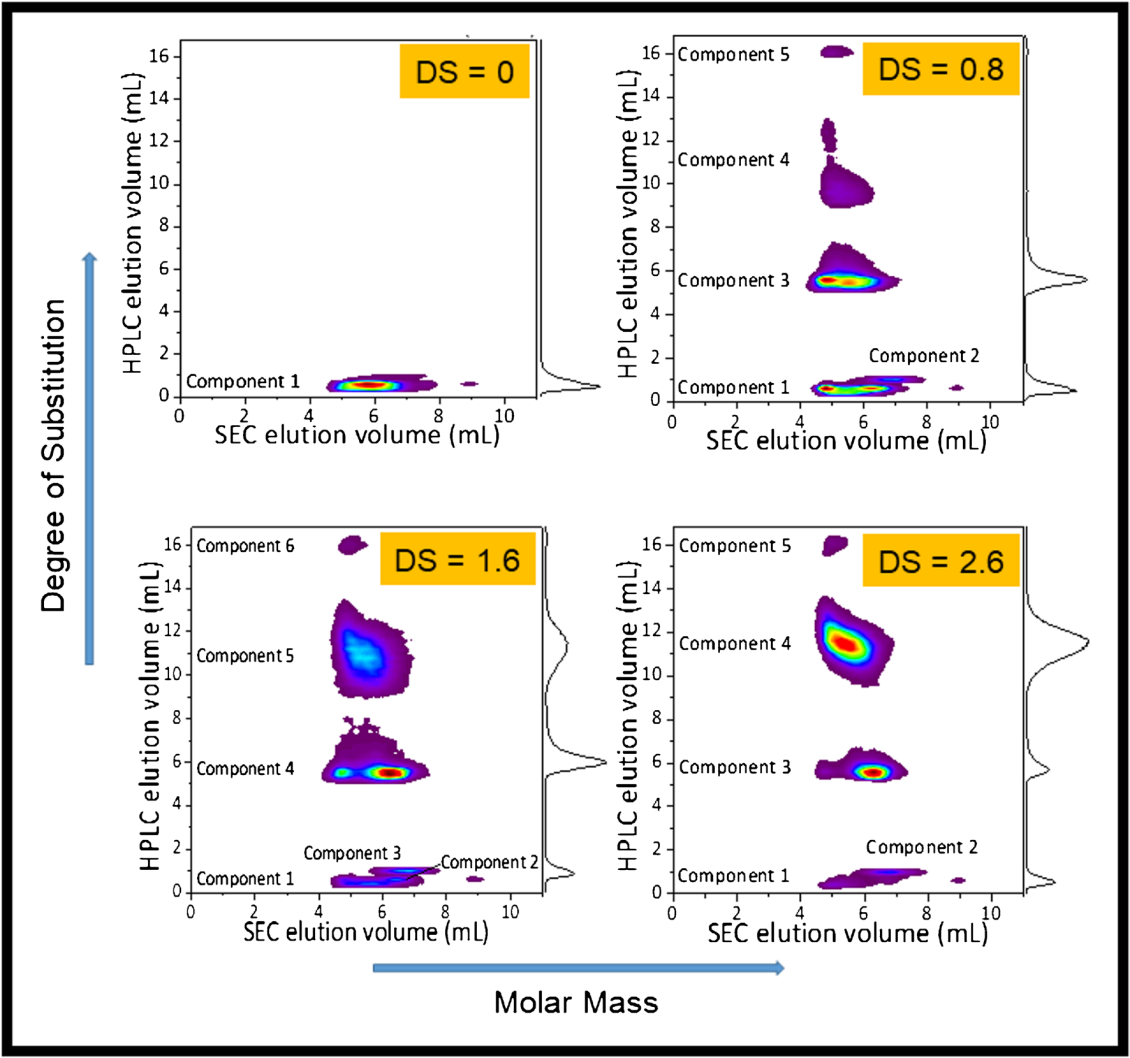
Separation of acrylate-modified HA according to degree of substitution (DS) and molar mass using comprehensive 2D-LC, 1st dimension: C_8_-silica, stepwise gradient ranging from 100% water to 50/50 v/v ACN-water, 2.^nd^ dimension: Suprema, 60/40 v/v ACN-water with 0.02 M NH_4_Ac (adapted with permission from Viktor et al. [30])

Some representative 2D-LC diagrams are presented in Fig. 7. It is seen that the hyaluronic acid derivatives are broadly distributed with regard to both chemical composition and molar mass. Fractions with different degrees of substitution are identified, and their molar mass distributions are determined. The study proved that comprehensive 2D-LC is a powerful approach to reveal the complex nature of hyaluronic acid and its derivatives.

## Multidimensional analysis of lignin derivatives

**Fig. 8:**
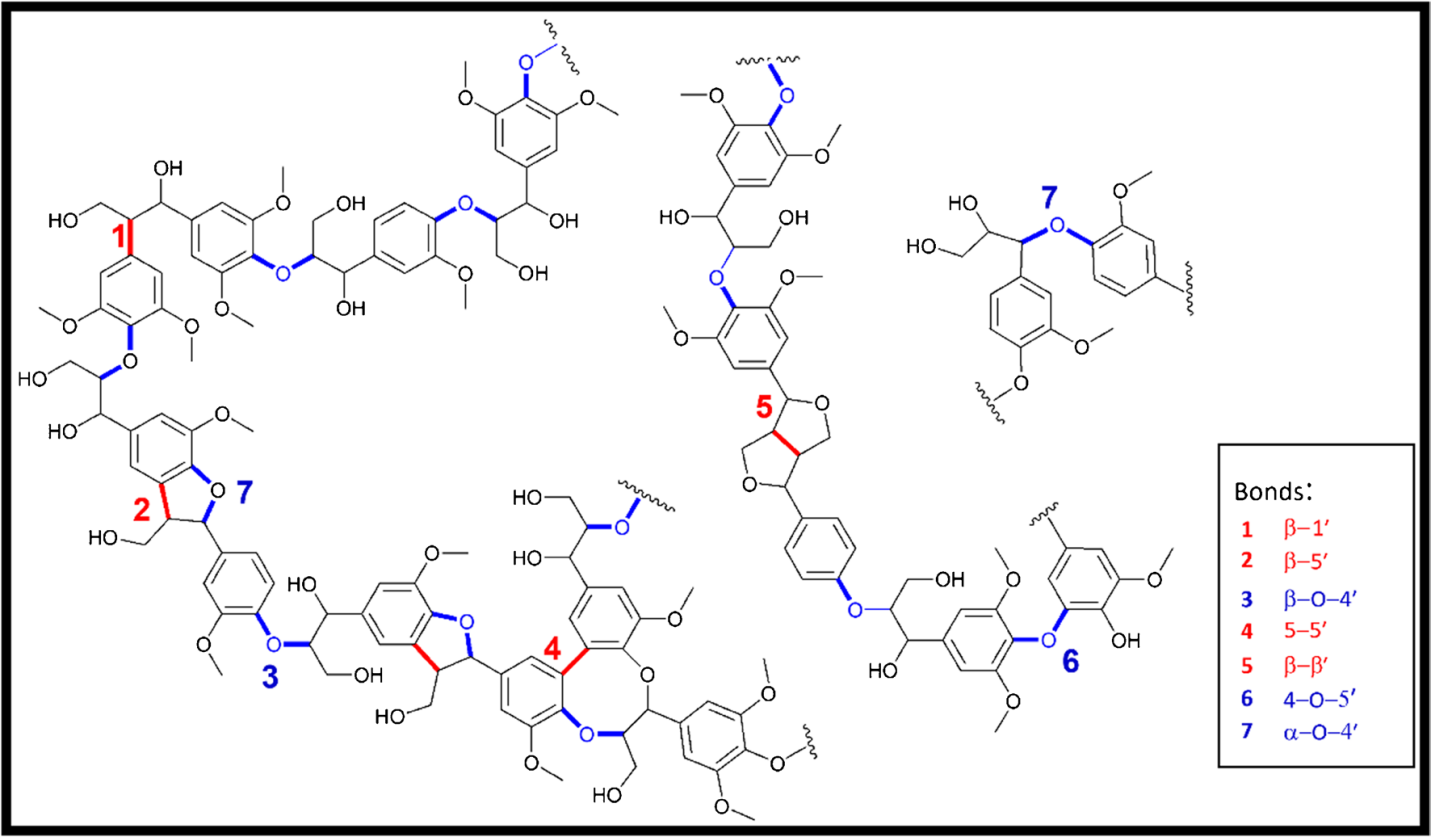
Main sub-structures present in lignin: (1) open β–1′ linkage, (2) phenylcoumaran structure with β–5′ and α–O–4′ linkages, (3) β–O–4′ linkage, (4) biphenyl structure formed by the 5–5′ linkage, (5) resinol structure with β–β′ linkages, (6) diaryl ether structure with the 4–O–5′ linkage, and (7) α–O–4′ linkage

Lignin is an extremely complex aromatic biopolymer with a unique composition and structure. It is composed of three basic phenyl propane building blocks (monolignols), namely coniferyl alcohol (G-unit), sinapyl alcohol (S-unit), and *p*-coumaryl alcohol (H-unit) (see Fig. 8). Though highly complex, there is increasing interest in lignin, because as the most abundant aromatic biopolymer, it has the potential to reduce our dependence on non-renewable feedstocks, such as crude oil as a source of aromatic compounds.

The lignin biorefinery can be divided into three interlinked technological processes: (1) lignocellulosic biomass fractionation, (2) lignin depolymerization, and (3) upgrading of products to specific chemicals, where depolymerization is seen as a suitable pathway to value-added low molar mass compounds [31, 32].

The characterization of lignin derivatives is a challenging task due to the very complex structure of these materials. Different advanced analytical methods have been used including FTIR spectroscopy [33], liquid chromatography [34], NMR spectroscopy [35], and even comprehensive two-dimensional liquid chromatography [36].

Various depolymerization methods have been developed to convert lignin to low molar mass compounds. Of these, lignin oxidative degradation presents numerous advantages as a sustainable route to aromatic monomers with aldehyde, carboxylic, hydroxyl, vinylic, and methoxy functionalities. Various experiments have been performed. According to our research, the combination of DMSO/HBr is an excellent approach combining mild experimental conditions with a large variety of valuable degradation products to be formed [37].

**Fig. 9:**
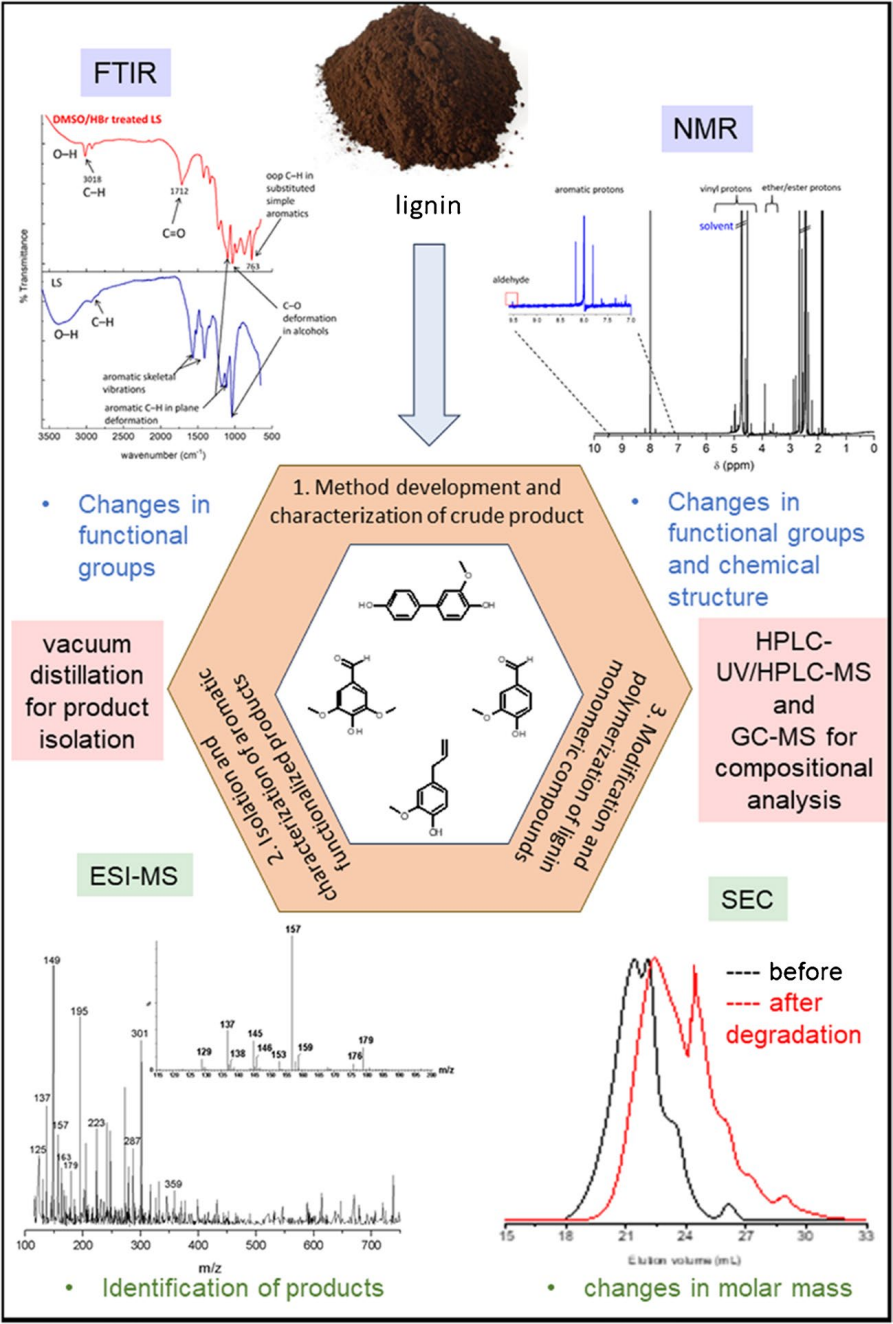
Analytical protocol and suitable techniques for the comprehensive analysis of depolymerized lignin

Lignin depolymerization leads to complex mixtures of monomeric and oligomeric compounds that can be analyzed only by the combination of different advanced analytical methods following the LEGO approach (see Fig. 3). The degradation in molar masses was followed by size exclusion chromatography; selected fractions were collected and subjected to a range of advanced analyses including high-resolution NMR, GC–MS, and HPLC–MS (see Fig. 9 for a schematic presentation).

**Fig. 10:**
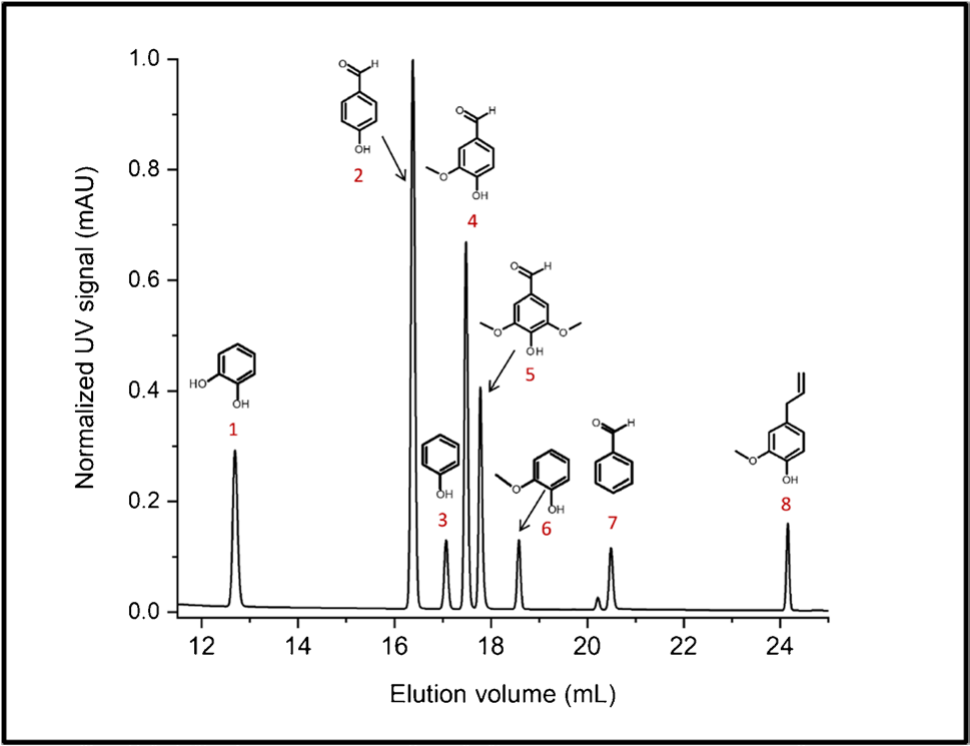
Separation and analysis of different monomeric compounds by HPLC–UV-ESI–MS; stationary phase: C_18_-modified silica, mobile phase: linear gradient from 98/2 v/v water-acetic acid to 98/2 v/v methanol–water

In a first step of the analysis of depolymerized lignin, bulk analysis was conducted. The decrease in molar mass was monitored by SEC and it was proven that the oxidative degradation leads to significantly reduced molar masses. Molar mass analysis of lignins is quite challenging because lignin solutions are known to show fluorescence. This has to be accounted for when spectrometric detection is used. In the present case, a differential refractometer was used and the shift in elution volume was taken as proof for the changes in molar mass. In the lower molar mass range, monomers and lower oligomers were detected. Information on the formation of new structures was obtained by FTIR and ^1^H-NMR which showed the presence of various carbonyl groups and aldehydes. ESI–MS was used to identify specific monomeric degradation products including vanillin, eugenol, hydroxycinamaldehyde, and others. The most feasible way to identify specific compounds, however, was via HPLC–UV-ESI–MS (see example in Fig. 10).

In further studies, the crude oxidation products were distilled and different monomeric compounds isolated. These compounds were quantified and the mass balance of the oxidative degradation process was evaluated [37].

**Fig. 11:**
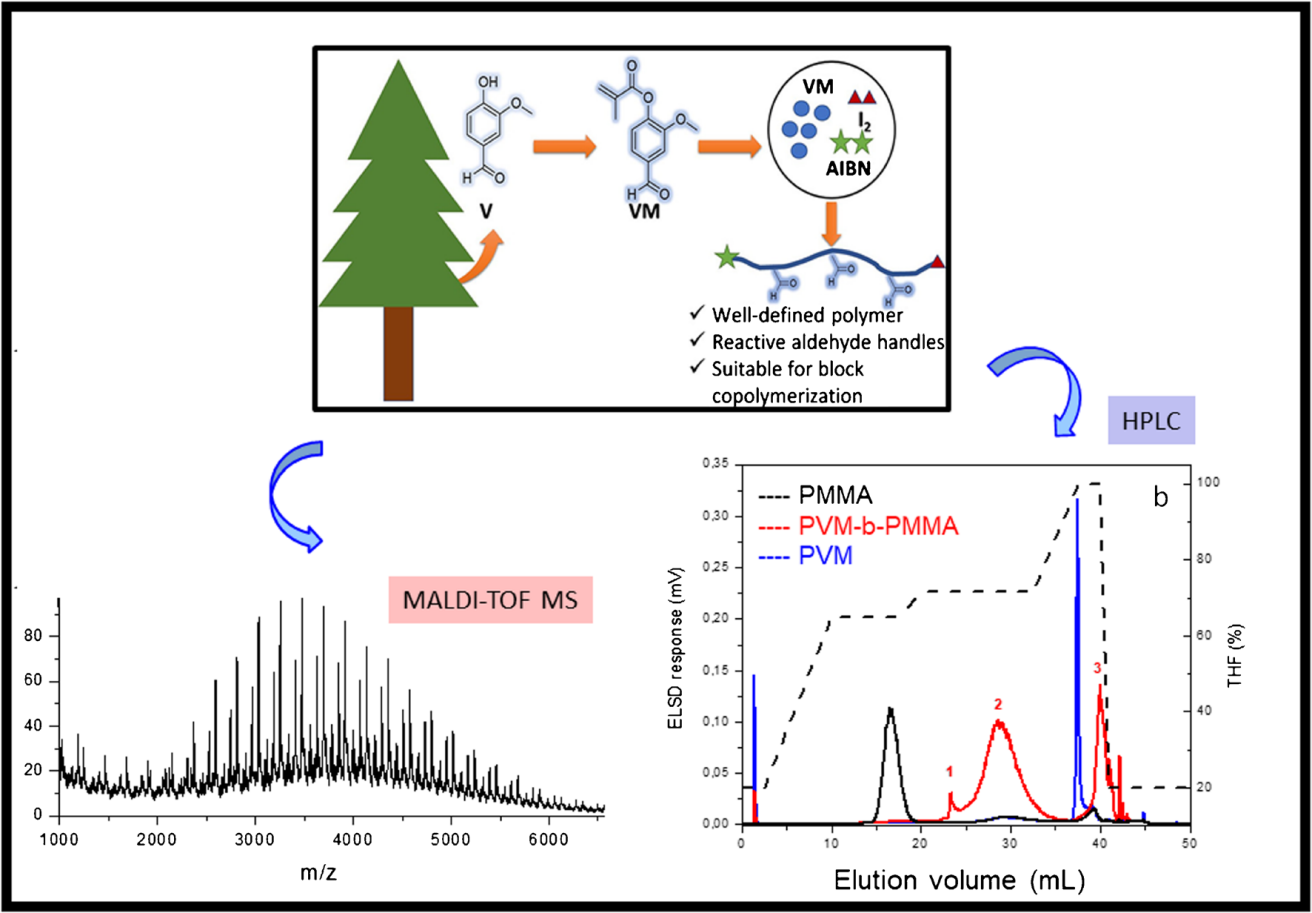
Derivatization of vanillin derived from lignin degradation to vanillin methacrylate followed by polymerization using RITP and suitable analytical protocols for structure elucidation (adapted with permission from Pfukwa et al. [38])

A further step towards valorization of lignin-derived compounds was taken by polymerizing these compounds using reverse iodine transfer polymerization (RITP) [38]. In this work, vanillin methacrylate (VM), an aldehyde-functionalized monomer synthesized by the methacrylation of vanillin (a lignin degradation product), was polymerized via RITP (see Fig. 11).

The RITP process involves the use of molecular iodine to control polymerization. The process is characteristically divided into two periods: (1) the inhibition period, where alkyl iodide chain transfer agents are generated from the reaction between an initiator (typically AIBN) and molecular iodine and (2) the polymerization period which is governed by a degenerative chain transfer mechanism. The influence of various reaction parameters including the polymerization solvent, the initiator-to-iodine ratio, and the polymerization temperature on the induction (or inhibition) period and the molar mass were investigated by NMR spectroscopy and SEC. The formation of polymer chains was also confirmed by MALDI-TOF mass spectrometry. To demonstrate the “livingness” of the polymerization process, PVM was chain-extended with methyl methacrylate, and the formation of block copolymers was confirmed by HPLC.

## Multidimensional analysis of wood extract tannins

The polyphenol family of vegetable tannins includes hydrolysable and condensed tannins [39]. The term “hydrolysable tannins” originates from the formation of ellagic and/or gallic acid as hydrolysis products of the corresponding tannins. The transformation of hides and skins into leather in the tanning process is the main use of these materials. For this purpose, chestnut and quebracho are the two most commonly used commercial tannin extracts, with chestnut in particular being used for tanning of leather soles. The interesting biological activities of these extracts include anti-cancer [40] and anti-HIV properties [41], and the treatment of diabetic complications [42].

**Fig. 12:**
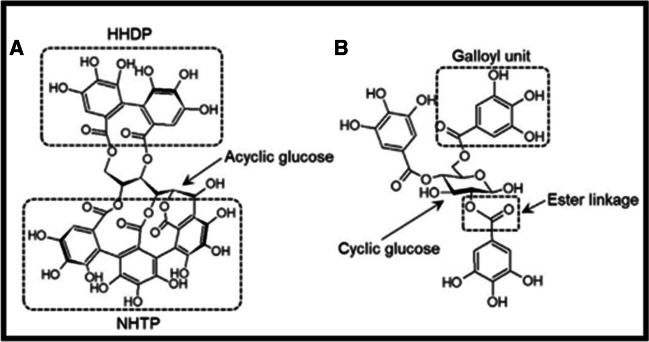
Representative structures of the two classes of hydrolysable tannins found in chestnut: (**A**) an ellagitannin (vescalagin) and a (**B**) gallic acid derivative (1,3,6-trigalloylglucose). HHDP, hexahydroxydiphenoyl; NHTP, nonahydroxytriphenoyl

The two main classes of hydrolysable tannins are ellagitannins and gallic acid derivatives. Ellagitannins contain a hexahydroxydiphenoyl (HHDP) unit being formed by the oxidative C–C coupling of adjacent galloyl units to a glucose core. The nonahydroxyterphenoyl (NHTP) group is also esterified to the polyol core of some ellagitannins (see Fig. 12). Ellagic acid is produced by hydrolysis of ellagitannins due to spontaneous lactonization or intramolecular esterification. Accordingly, hydrolysable tannins as complex multicomponent mixtures require comprehensive analysis in a multidimensional approach.

**Fig. 13:**
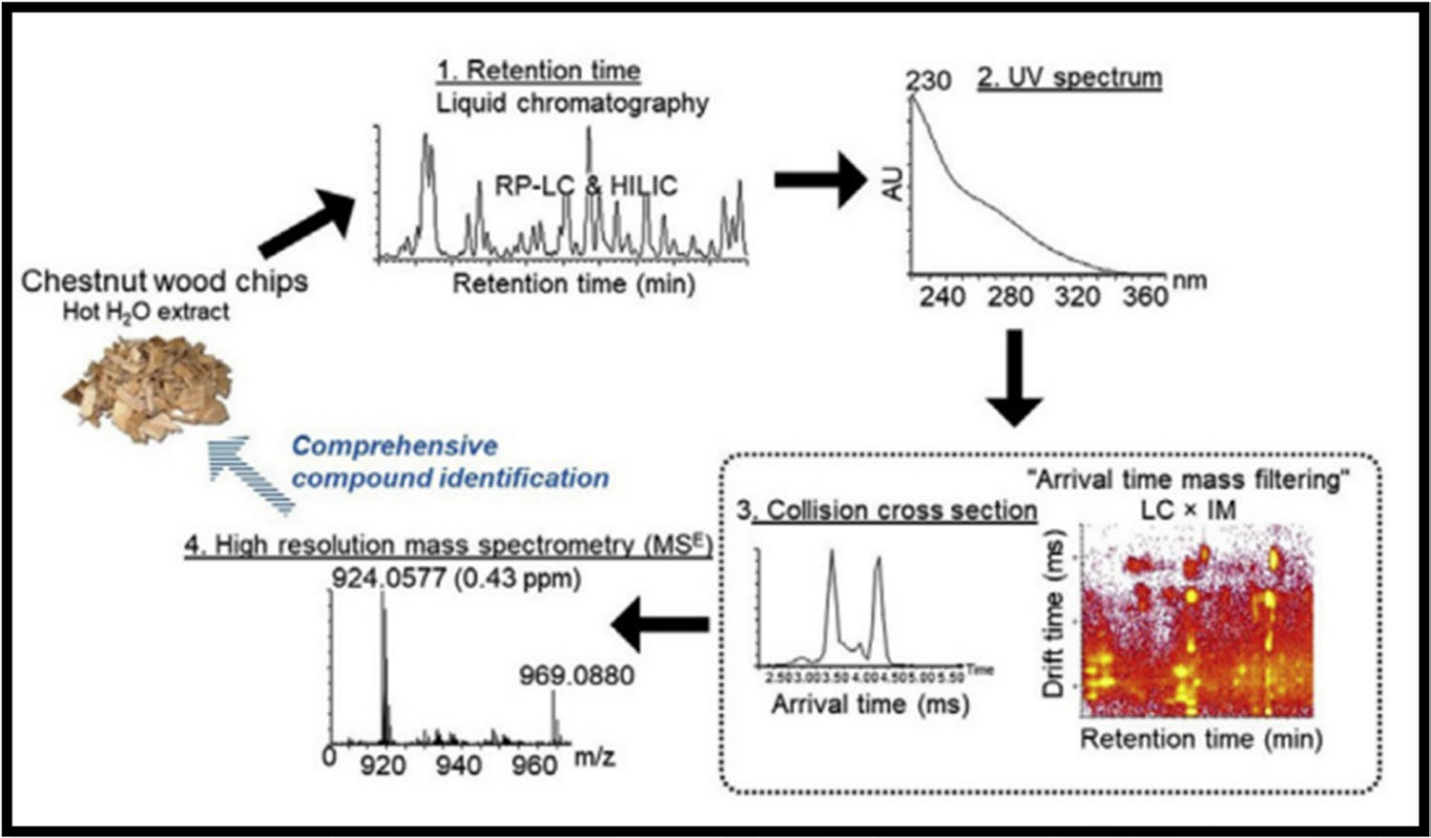
General scheme for the comprehensive analysis of chestnut tannin extracts using the multidimensional combination of HPLC and HR-MS (adapted with permission from Venter et al. [43])

The approach is based on reversed phase LC (RP-LC) and hydrophilic interaction chromatography (HILIC) separations coupled to ion mobility (IM) and high-resolution mass spectrometry (HR-MS). The application of this approach to the analysis of chestnut (*Castanea sativa*) tannin extracts has been demonstrated by Venter et al. [43]. A total of 38 molecular species, including a large number or isomers, were identified based on HR-MS and UV spectral information as well as retention behavior in both LC separation modes. In total, 128 and 90 isomeric species were resolved by RP- and HILIC-LC-IM-TOF–MS, respectively. The combination of low- and high-collision energy mass spectral data with complementary chromatographic separations allowed for the identification of twenty molecular species, comprising 78 isomers, in chestnut. Ion mobility resolved six new dimeric and trimeric vescalagin conformers with unique arrival (drift) times, which were not separated using either RP-LC or HILIC. The general scheme of this comprehensive approach is presented in Fig. 13. Further work on other types of tannins and comprehensive three-dimensional LC × LC × ion mobility spectrometry separation has been published by the same group [44, 45].

## Outlook

The comprehensive characterization of complex bio-based and biodegradable polymers requires a toolbox of advanced analytical techniques that are capable of addressing the different parameters of molecular heterogeneity including molar mass/molecular size, chemical composition, microstructure, and molecular topology. As all molecular parameters are distributed properties, different methods of chromatographic fractionation must be used in addition to standard spectroscopic methods such as FTIR, NMR, and mass spectrometry. For a number of selected applications, it is demonstrated that a maximum depth of information is obtained when different chromatographic modes are combined in multidimensional chromatographic setups. Other most promising experimental protocols relate to the combination of selective HPLC methods and information-rich detectors (FTIR, NMR, MS) preferably in online modes. It has been shown that the combination of 2D-LC and MS^n^ methods allow for the comprehensive analysis of complex samples that are distributed regarding molar mass, chemical composition, and microstructure. In conclusion, the work presented here clearly indicates that, in future, for the comprehensive analysis of bio-based and biodegradable polymers, the LEGO approach of combining multiple advanced analytical methods is the most suitable strategy.
